# Chondromalacia patellae: current options and emerging cell therapies

**DOI:** 10.1186/s13287-021-02478-4

**Published:** 2021-07-18

**Authors:** Weitao Zheng, Hanluo Li, Kanghong Hu, Liming Li, Mingjian Bei

**Affiliations:** 1 Hubei Provincial Key Laboratory of Industrial Microbiology, Sino-German Biomedical Center, National “111” Center for Cellular Regulation and Molecular Pharmaceutics, Hubei University of Technology, Wuhan, 430068 Hubei Province China; 2 Shanxi Yinmei Technology Co., Taiyuan Economic and Technological Development Zone, Room 301, No. 8, East Street, Taiyuan, China; 3 Department of Orthopedic Surgery, Emergency General Hospital, Xibahenanli29, Chaoyang dis, Beijing, 100028 China

**Keywords:** Chondromalacia patellae, MRI, Cell therapy, Chondrocyte implantation, Mesenchymal stem cells

## Abstract

Chondromalacia patellae (CMP), also known as runner’s knee, typically occurs in young patients, which is characterized by anterior knee pain (AKP) that is associated with visible changes in patellar cartilage. The initial pathological changes include cartilage softening, swelling, and edema. CMP is caused by several factors, including trauma, increased cartilage vulnerability, patellofemoral instability, bony anatomic variations, abnormal patellar kinematics, and occupation hazards. CMP may be reversible or may progress to develop patellofemoral osteoarthritis. Quadriceps wasting, patellofemoral crepitus, and effusion are obvious clinical indications. Additionally, radiological examinations are also necessary for diagnosis. Magnetic resonance imaging (MRI) is a non-invasive diagnostic method, which holds a promise in having the unique ability to potentially identify cartilage lesions. Modalities are conventionally proposed to treat cartilage lesions in the PF joint, but none have emerged as a gold standard, neither to alleviated symptoms and function nor to prevent OA degeneration. Recently, researchers have been focused on cartilage-targeted therapy. Various efforts including cell therapy and tissue emerge for cartilage regeneration exhibit as the promising regime, especially in the application of mesenchymal stem cells (MSCs). Intra-articular injections of variously sourced MSC are found safe and beneficial for treating CMP with improved clinical parameters, less invasiveness, symptomatic relief, and reduced inflammation. The mechanism of MSC injection remains further clinical investigation and is tremendously promising for CMP treatment. In this short review, etiology, MRI diagnosis, and treatment in CMP, especially the treatment of the cell-based therapies, are reviewed.

## Introduction

The knee joint is a tricompartmental structure comprising the patellofemoral (PF) joint and medial and lateral tibiofemoral (TF) joints. Anterior knee pain (AKP) is usually focused on TF disorders, while PF has rarely been concerned. PF disorders commonly cause AKP and giving way, which is usually aggravated by squatting, running, stair climbing and, other activities [1]. Among the most common PF disorders leading to AKP are chondromalacia patellae (CMP), lateral patellar compression syndrome (LPCS), and osteoarthritis (OA) [2]. CMP, also known as runner’s knee, is a common cause of AKP among young people, especially young women who love sports [3], and is characterized by AKP that is associated with visible changes in patellar cartilage. Sometimes, it is also called a catch-all phrase to describe PF pain with or without documented chondral abnormality. The thickness and integrity of the covered hyaline cartilage determine the health of the patella.

The normal appearance of the patellar hyaline cartilage is bluish-white, smooth, glistening, and resilient. The initial pathological change in CMP is that the cartilage becomes dull or even slightly yellowish-white, and turns soft, swollen and edema in the early stage [4, 5], Characteristically, the lesion is usually in the middle of the medial patellar facet, or just distal to that point, and is about half an inch or more in diameter [5], and followed by the fibrillation, fissure, fragmentation, or erosion of the cartilage at the advanced stage [5–7]. In 1906, Budinger et al. firstly reported the pathological changes of patellar cartilage, and then Kelly et al. considered this pathological phenomenon as CMP [7]. The original definition of CMP is the softening of the patellar bone cartilage layer or a phenomenon that the cartilage layer of the patella is no longer as dense as it used to be in the healthy state. “Chondromalacia” is derived from the Greek words. Chrondros means cartilage and malakia means softening [8]. Ralph Edward Outerbridge et al. [5] considered that the pathological change process of CMP can be classified into four grades: in grade1 cartilage lesions emerged as softening and swelling, edema; in grade 2, there are fragmentation and fissuring in an area half an inch or less in diameter; grade 3 is the same as grade 2 but an area more than half an inch in diameter is involved; and in grade 4, there is cartilage denudation, erosion of cartilage down to the subchondral bone, and proliferation. In terms of the Asian population, Ye et al. [9] considered that grade 1–2 is the CMP early stage, whereas grade 3–4 is the characterization as the advanced stage. Meanwhile, they speculated that the patellar cartilage has the ability to repair itself in the early CP (stage 1–2); however, the cartilage lesions in the advanced CMP (stage 3–4) has developed into patellofemoral joint osteoarthritis (PFJOA), and then patellar cartilage has no real self-repair ability [9]. Similarly, another report also considered that CMP could be reversible or might proceed to PFJOA [10]. Chondrocyte replication suggests a healing ability in early cases following treatment that changes the load by affecting cartilage. Therefore, interventions in an early stage may be more promising, whereas the early stage has to be identified first. The identification of CMP seems to play an important role in the early and maybe even the preclinical stage of PFJOA.

## The etiology of CMP

Although the etiology of CMP is complex, several factors, such as trauma (e.g., direct to patella), increased cartilage vulnerability (congenital, post-arthrotomy/casting rehabilitative period, etc.), patellofemoral instability (dislocation, subluxation, etc.), bony anatomic variations (congenitially flattened lateral femoral condyle, osteochondral ridge, etc.), abnormal patellar kinematics (patella alta, valgus knees, excessive laterally placed tibial tubercle, etc.), and occupation hazards (athletic trainees and army, jobs requiring excessive kneeling and squatting, etc.), are involved in the CMP etiology. Among the causes of CMP, subluxation is probably the most common and the most frequently missed since there has been a frank patellar dislocation [11].

In the recent decades, researchers have found several new findings. In a study of 301 patients with knee pain, the researchers investigated the relationship between PFJ morphology and CMP using MRI. To evaluate the severity of the patella, the researchers measured the sulcus angle, trochlear depth, and patellar angle, and evaluated the patellar tilt by using the lateral patellar tilt angle. The results showed that the lateral patellar tilt angle and trochlear depth in patients with CMP were significantly decreased, while the sulcus angle was significantly higher, and the correlation between patella angle and CMP was not observed [12]. Another MRI study of 200 patients with knee pain also found that the CMP patients had lower lateral patellar tilt angle, lower trochlear depth, and higher sulcus angle. It also suggested that the trochlear sulcus angle to trochlear depth ratio could be used as a marker in the early period of CMP development [13]. In addition to lateral patellar tilt angle and trochlear depth, tibial slope and patellar height are also important factors related to the probability of CMP [14].

Another notable factor associated with CMP is the subcutaneous knee fat thickness. The study by Hong Kuan K et al. assessed the association between obesity and CMP, they found that the subcutaneous knee fat thickness of 33 CMP patients identified by MRI was significantly higher than that of the normal group, and a significant correlation between subcutaneous knee fat thickness and grades of CMP was also observed. In addition, the female patients had a thicker subcutaneous knee fat and more serious CMP than male patients [15] This correlation had also been confirmed in the study mentioned above [12]. It can be concluded that the increased subcutaneous knee fat thickness is associated with the occurrence of CMP along with high sulcus angle, low trochlear depth, and low patellar tilt angle.

## Diagnosis

Physical signs of AKP, such as effusion, quadriceps wasting, and retropatellar crepitus, have been claimed to be more informative in the diagnosis of CMP. However, none of these signs is considered specific for CMP [6]. Reliable diagnosis of CMP requires the exclusion of diseases that can also lead to the symptoms of AKP syndrome, such as patellar malalignment, excessive lateral patella pressure, osteochondral injury, meniscal tear, Hoffa’s syndrome, and Synovial plica, as there are great differences in the treatment of these diseases, especially in the choice of surgical treatment. The lower sensitivity and specificity of X-ray is a limitation for diagnosis of early stages of CMP; radiographs have not proven to be useful in the diagnosis of CMP until the advanced stages, such as extensive cartilage loss, joint space loss, and associated changes of sclerosis and cystic in the subchondral bone. Arthrography combined with radiographs may reveal imbibition of contrast into areas of chondromalacia, but again sensitivity is low. CT arthrography may successfully confirm focal areas of cartilage irregularity or loss, but also until the advanced stages [16]. Arthroscopy can achieve a reliable diagnosis, as it allows a good view of the PFJ [17]; however, there is no correlation between the severity of the CMP and the clinical symptoms of AKP syndrome. Thus, these symptoms should not be used as an indication for knee arthroscopy. Moreover, arthrography, as an invasive diagnostic method, as well as being a modality, is usually used only when the advanced stages of disorders. Magnetic resonance imaging (MRI), which is a non-invasive diagnostic method, holds a promise in having the unique ability to potentially detect cartilage lesions, as well as the internal disarrangement in cartilage before macroscopic morphological cartilage loss.

It would be helpful if MRI could confirm the diagnosis of CMP, which is a more comfortable procedure, as well as with a lower risk of complications, than that associated with diagnostic arthroscopy for the patient. MRI has gradually replaced arthroscopy as a non-invasive and reliable means to identify CMP [18, 19]. An earlier study comparing arthroscopy and 1.0-T MRI showed that MRI had a higher detection rate for more severe CMP [18]. The study used the four grades rating system of Shahriaree to measure the severity of cartilage lesions. For the 56 patients with anterior knee pain, 25 patients were diagnosed CMP positive using arthroscopy, among which 17 patients were defined as grade II and III CMP, and the diagnostic accuracy was 68% in the CMP positive patients. Meanwhile, 20 patients were diagnosed with CMP using MRI, in which 18 patients were diagnosed as grades II and III, with 90% diagnosis accuracy in positive patients. In addition, none of the 36 negative patients who did not have CMP, as identified by MRI, were identified as CMP positive severer than grade II by arthroscopy; however, among the 31 negative patients identified by arthroscopy, 2 patients were diagnosed as grade III CMP by MRI [18]. However, the severity of CMP is difficult to correlate with clinical symptoms, such as AKP syndrome, it is unclear whether MRI can benefit the accurate diagnosis of CMP in patients with AKP. Unsurprisingly, therefore, the reported accuracy of MRI for cartilage lesions in CMP varies widely in the literature. Previous studies showed that the sensitivities ranged from 26 to 100%, the specificity ranged from 50 to 94%, and the diagnostic accuracy ranged from 77 to 90% [20–25]**.** These studies had varied widely with regard to the imaging methods, patient samples, and grading systems utilized, which probably explains the different results.

Along with the progress of medical iconography, MRI can present images with higher resolution and clarity in a faster and more accurate way. Most notably, in a recent study, by using 3.0-T MRI system, researchers developed a more detailed grading system on the basis of the arthroscopic cartilage injury grading system developed by the International Cartilage Research Society [19]. Under grade 1, it was further divided into 1A, 1B, and 1C. Under each of the grades 2, 3, and 4, there were four sub-classifying grades of A, B, C, and D. in addition, any grade 4 lesion with subchondral fibrocystic bony changes are classified as grade 5 [19]. More detailed classification of patellar cartilage injury will enable us to make a better diagnosis of CMP, and more precisely predict the prognosis and clinical outcomes of patients.

## Intervention

CMP may be reversible or may proceed to PFJOA [10]. Unfortunately, the cartilage is known to have no self-repair ability in the advanced OA. Therefore, early diagnosis and interventional treatment for CMP are more important and efficient for managing this disorder.

### Conservative treatment

As we all know, nonoperative intervention seems to as the initial treatment for all patients experiencing AKP, including mainly activity restrictions or rest and nonsteroidal anti-inflammatory drugs when necessary. Additionally, before surgeries were considered, patients were always instructed and encouraged to perform exercises supervised by a physiotherapist for strengthening the quadriceps muscle, reducing Q angle, and crepitation. The simplest effective procedure that avoids quadriceps dysfunction and fibrosis is a distal patellar tendon medial realignment with lateral release and medial reefing of the quadriceps expansion. Bakhtiary et al. [26] and Petersen et al. [27] showed that strengthening the quadriceps muscle by different exercises markedly alleviated the AKP in early CMP patients.

### Surgical treatment

When CMP progresses to the end stages and conservative care fails, surgical treatment can be an effective alternative, such as patellar cartilage excision, shaving, drilling, or proximal soft tissue and distal bony patellar realignment surgery; however, the choice of the best procedure is difficult as each measure has its own relative indications and limitations, especially in the grade of patellar cartilage lesions and the patients’ age. Patellectomy includes partial patellectomy and total patellectomy, but this must be performed only when the patient has excellent quadriceps function before surgery and is motivated to exercise after surgery. Total patellectomy is a radical management for CMP, which has greater damage to the surrounding ligaments and quadriceps femoris [28] as well as changing the leverage effect of extensor muscle, instability of extensor tendon, acute rupture of the patellar tendon and other complications may occur in the later stage compared with partial patellar resection [29, 30]. So, partial patellectomy was usually employed in treating CMP. Tibial tuberosity surgeries, consisting mainly of the tibial tuberosity osteotomy, tibial tuberosity anteversion, and tibial tuberosity elevation, by restoring the biomechanical force line of the patellofemoral joint could improve joint function, but it will promote the degeneration of PFJ to a certain extent [31]. Harrington et al. reported that the McKeever patellar resurfacing prosthesis showed a beneficial long-term effect in the treatment of severe CMP, which is usually as a salvage procedure for advanced PFJOA [32]. However, due to the existence of many complications, such as patellar tendon lesions, secondary patellar fracture, ischemic necrosis, instability of PFJ, and prosthesis loosening, this modality has been gradually neglected [33]. Arthroscopy could smooth the fibrillated and traumatized areas of articular cartilage, which is common used in grades II, III, and IV CMP. However, treatment with arthroscopy is indicated in < 10% of patients [34]; moreover, the initial treatment of CMP require a period of rehabilitation, and furthermore, as a reliable diagnostic method, if the examination does not found any arthroscopically treatable injury, it may seem to be a costly diagnostic method unnecessarily consuming our limited health-care resources [18]**.** Meanwhile, arthroscopy causes short-term functional disability, pain, and stress and involves risks associated with anesthesia and surgery [18].

### Autologous chondrocyte transplantation

All measures mentioned above are not aimed at the damaged cartilage, but to alleviate symptoms, maintain function, and minimize disability rather than regenerate articular cartilage, considering that CMP is characterized by softness, of cartilage swollen and edema in the early stage, and followed by fibrillation, fissure, fragmentation or erosion of the cartilage at an advanced stage, none of them have emerged as fundamental treatment.

In recent two decades, large numbers of studies in treating OA have focused on the level of cell therapy. Cell transplantation is an emerging therapeutic management for OA treatment, consisting mainly of the utilization of autologous chondrocytes and relevant cartilage tissue [35, 36]. Similar to OA, present reviewers agree that CMP is a mesenchymal disease, that is to say, the positive therapeutic effect at cell level on OA will also be beneficial to CMP. The emerging cell therapies, including autologous chondrocyte transplantation and MSCs injection, are becoming new treatment options for CMP patients.

In 1994, Brittberg et al. first reported autologous chondrocyte implantation (ACI) [37]. They performed ACI in 23 patients with full-thickness cartilage defects of the knee joint. Two years after implantation, 14 out of 16 patients with femoral condylar implantation achieved satisfactory results. However, of the 7 patellar implantations, only 2 had good or excellent results [37]. In fact, there have been many reports on the treatment of cartilage injury by autologous chondrocyte implantation [38–41]. In October 2009, ChondroCelect, an autologous chondrocyte product from TiGenix (which was acquired by Takeda in 2018), was approved for marketing in October 2009. An early study by Simon Macmull et al. performed autologous chondrocytes implantation showing positive clinical outcomes on 48 patients with CMP, they found that the subjective pain score and objective function scores were significantly improved over a mean follow-up period of 40.3 months [42]. In their study, the patients’ own chondrocyte cells were cultured in vitro for 4–6 weeks and then implanted back. Of the 48 patients, 25 received the ACI method and 23 received the Matrix-assisted Chondrocyte Implantation (MACI) method. In the ACI method, the cultured cells were directly infiltrated under a collagen I/III membrane that was previously sutured to the cartilage defect. In the MACI method, the cultured cells were pre-seeded on a type I/III collagen membrane at the density of 1 × 10^6^/cm^2^ and then adhered to the cartilage defect with fibrin glue. Finally, it was confirmed that the cartilage lesions in CMP patients responded well to chondrocyte implantation, moreover, the MACI method had a better treatment outcome than the ACI method, additionally, the MACI procedure is technically easier and less time consuming [42]. In December 2016, Vericel’s MACI, for the treatment of knee cartilage injury, was also approved by FDA, and it was the first cell therapy product approved by FDA by using a porcine collagen membrane scaffold. There were not many studies on how the implanted chondrocytes interact with the cartilage tissue in vivo, but it seems that exogenous chondrocytes form new hyaluronic cartilage through proliferation, migration, and secretion of extracellular matrix to repair [43, 44]. Although autologous chondrocytes as the main cell type in cartilage may provide a safe and efficacious method, chondrocyte implantation has inherent drawbacks of limited availability, de-differentiation, and function loss during culture, such as chondrocyte dedifferentiation in vitro expansion that might result in fibrocartilage rather than hyaline cartilage [45]. Moreover, an additional surgical procedure may lead to further cartilage injury and degeneration [37, 45]. Therefore, the application of MSCs for CMP therapy has attracted much more attention from scientific investigators.

### MSCs injection

In recent years, autologous mesenchymal stem cells (MSc) have become the most important source of adult stem cells in basic research and clinical application. MSCs have significant advantages in regenerative medicine due to its self-availability, pluripotent differentiation, paracrine nutrition effect, immune immunity, non-tumorigenicity, and safety [46, 47]. There are abundant sources of MSCs in the human body, mainly in the bone marrow. MSCs also exist in non-marrow parenchyma tissues, including peripheral blood [48], adipose tissue [49], wisdom teeth [50], deciduous teeth [51], synovial fluid [52], hair follicles [53], and in neonatal umbilical cord blood [54] and umbilical cord [55, 56].

The chondrogenic differentiation is among the minimal prerequisites for defining MSCs, and it is the base for tissue engineering procedures of generating articular cartilage [55]. Differentiation commitment of MSCs towards osteochondral lineages is determined by the mediation of Indian hedgehog (Ihh) and Wnt/ß-catenin signaling pathways, which osteoblastic progenitors differentiate into chondrocytes in the absence of ß-catenin [57]. The pre-requisite driving force towards the chondrogenic differentiation in vitro is the TGF-β superfamily [58]. Extracellular TGF-β superfamily promotes early and intermediate phase of chondrogenesis [59] via binding the TGF-β receptor type II, subsequently phosphorylating TGF-β receptor type I, which activates Smad 2/3 and 4signaling pathway, thereby activating Sox9 expression. Transcription factor Sox9 initiates early chondrogenesis by inducing the expression of collagen type II, alpha 1 (COL2A1), and other downstream genes Col I, Col II, Coll IX, and ACAN [10]. TGF-β superfamily was also reported to facilitate early chondrogenesis via inducing Runx2 (Runt-related transcription factor 2) and enhances the production of type II collagen and aggrecan, which are the main cartilaginous extracellular matrix [60].

MSCs are known to stimulate chondrocytes to proliferate and synthesize extracellular matrix [61, 62], and have demonstrated anti-inflammatory and immunomodulatory effects [63]. Previous studies have been reported that MSCs modulate inflammation and provide the environment for tissue regeneration either by directly secreting bioactive materials or by controlling cytokine and growth factor production from endogenous cells [64–66]. MSCs contributed to the repair of damaged articular cartilage through homing, engraftment, and production of cartilage matrix [67, 68] in OA models. Differentiation of delivered MSCs into chondrocytes appeared to be induced by the local environment of the homing site [68]. Barry et al. [69]. and Caplan et al. [64] considered that the potential mechanisms of MSCs for the treatment of cartilage lesions are believed in two ways. One is direct differentiation into chondrocytes, and the other is paracrine effects through secretion of bioactive materials should involve.

The first case of CMP treatment by using MSCs came from a Korean research group. The researchers isolated the patient’s own stromal vascular fraction which contains adipose-derived MSCs and injected it into the retro-patellar joints of three patients, mixed with platelet-rich plasma and hyaluronic acid. After 3 months of the treatment, the pain of 3 patients was reduced by 80–90%, and MRI showed that the damaged patellar cartilage tissue was almost completely restored compared with that before treatment [70]. However, it should be noted that patients in this study received multiple injections of platelet-rich plasma and hyaluronic acid or very low-dose dexamethasone on the 3^rd^, 7^th^, 14^th^, and 28^th^ days after the initial injection of the adipose-derived mesenchymal stem cell mixtures. In addition, without a control group also makes it hard to conclude that the possible treatment mechanism was solely due to adipose-derived mesenchymal stem cells, but most likely because of a comprehensive factor, just as the researchers analyzed by themselves [70]. Similar to OA, the reviewers agree that CMP is a mesenchymal disease, a condition in which the activity, phenotype, or mobilization of MSC population is altered, leading to an absence of repair and increased degeneration of cartilage [66, 69]. In OA, MSCs are depleted and have reduced proliferative capacity and reduced ability of differentiation [71]. Therefore, it would be beneficial if enough number of healthy and functional MSCs are provided to enhance self-repair or inhibit the progression of cartilage loss [72].

The positive role of MSCs in the treatment of knee joint cartilage injury and knee OA has no doubt. Moreover, in January 2012, South Korea had approved MEDIPOST’s CARTISTEM®, which is umbilical cord blood-derived mesenchymal stem cells, for the treatment of knee cartilage defects in patients with OA caused by degeneration or repetitive trauma. For the mechanism of cartilage repair induced by MSCs, more and more evidence tends to support the secretion role of MSCs rather than its differentiation capacity of directly differentiate into chondrocytes [73, 74]. Pleumeekers and others performed the co-culture of human adipose-derived MSCs or bone marrow MSCs with bovine chondrocytes in vitro, or implanted them into NMRI nude mice with cartilage incision along the central line of the spine, and found that human MSCs could promote chondrogenesis, and the new cartilage tissue only came from bovine chondrocytes [73]. The results of another phase I clinical trial by Tommy S de Windt et al. showed that the regenerated knee cartilage tissue was derived from the cells of receptors themselves after the implantation of allogeneic bone marrow MSCs, they demonstrated that Stem cell-induced paracrine mechanisms may play an important role in the chondrogenesis and successful tissue regeneration found [74]. Therefore, it is clear that the cartilage repair mechanism by MSCs is more likely because of their trophic effects and the induction of the regeneration of chondrocytes.

Recent animal reports have revealed the beneficial effect of MSCs on improving clinical symptoms and facilitating cartilage repair in OA [75–77]. Another study showed that intra-articular injection of human embryonic stem cell-induced mesenchymal stem cells (ESC-MSCs) significantly retarded cartilage destruction in a DMM OA mice model. Further in vitro studies illustrated that intra-articular injection of exosomes derived from ESC-MSCs successfully alleviated cartilage destruction by increasing collagen type II synthesis and decreasing ADAMTS-5 expression, which exerted a beneficial therapeutic effect on OA [78]. In Wang et al.’s [79] study, they demonstrated that the intra-articular injection of autologous human adipose-derived mesenchymal stem cells (haMSCs ) promote the regeneration of the articular cartilage in a rabbit OA model.

**Fig. 1:**
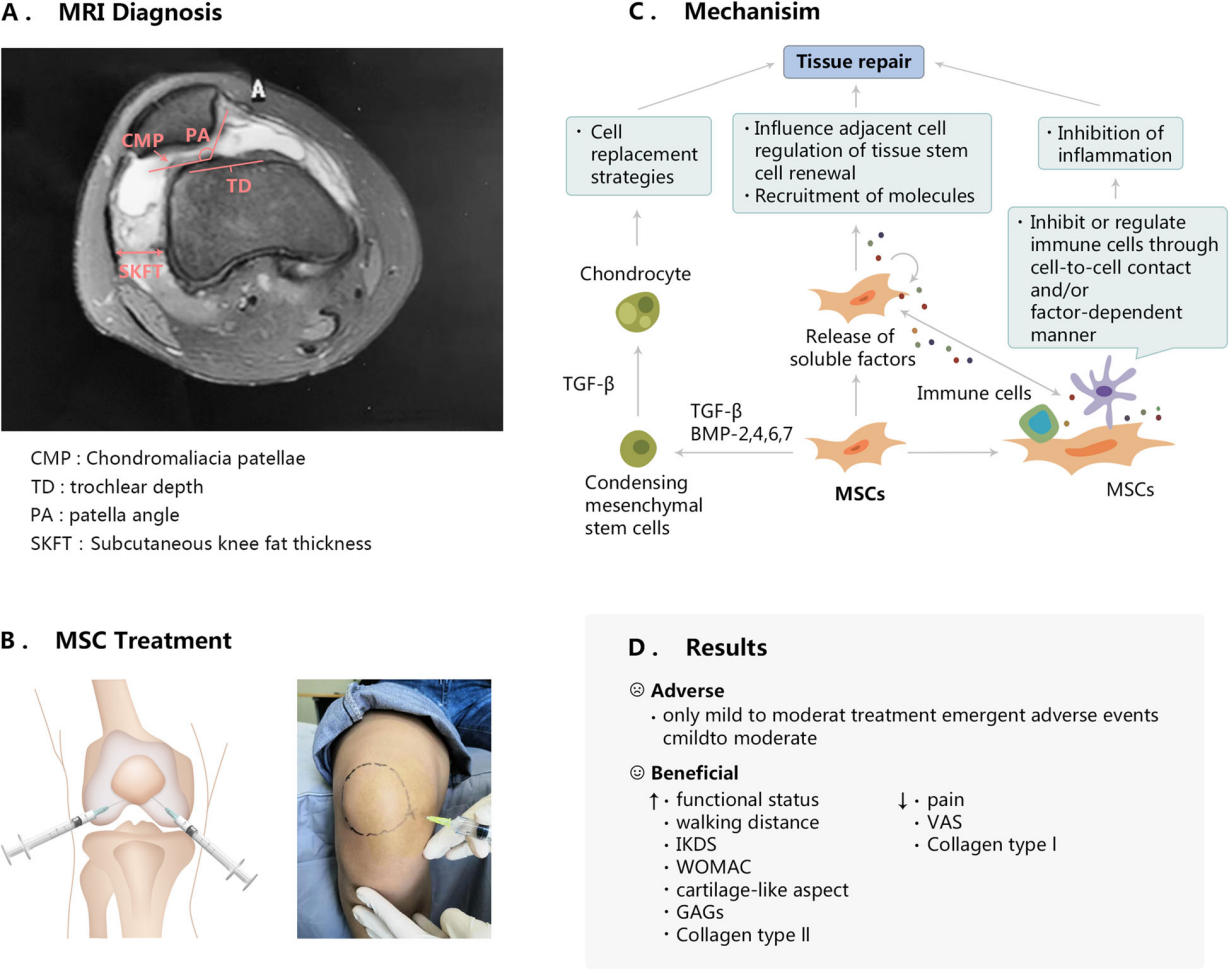
Schematic illustration of diagnosis (MRI) and intervention (MSC-based cell therapy) for CMP. **A** Representative MRI images for diagnosing CMP. **B** Demonstration of MSC injection into cavum articulare for treating CMP. **C** Three main mechanisms of MSC-based chondrogenic effects for CMP treatment: (a) MSCs differentiate into chondrocytes for cell replacement, (b) soluble factors released by MSCs act on the adjacent cells for tissue repair, and (c) MSCs inhibit immune responses via suppressing the proliferation of lymphocytes and inhibitory effects of the antigen presenting of dendritic cells. **D** Clinical indicators for evaluating the efficacy of CMP treatment using MSC-based cell therapies

Large quantities of preclinical evidences revealed the safety and efficacy of intra-articular injection of MSCs for the knee OA treatment. In another report from Wang et al.’s [79], they performed an intra-articular injection of haMSCs with different doses for the knee OA treatment, and during the procedure, three injections are provided and the patients were followed up for 96 weeks. They demonstrated that intra-articular injections of haMSCs had no adverse event and increasing cartilage volume in the knee OA, and haMSCs maintained the long-term improvement on symptomatic relief, function, and quality of life, which could be a promising novel treatment for knee osteoarthritis [79]. Similarly, Chris Hyunchul et al. [66] also found that there was no adverse event by using the intra-articular injection of autologous adipose tissue-derived MSCs (AD-MSCs) with different doses for treating knee OA. Meanwhile, they have confirmed that the Western Ontario and McMaster Universities Osteoarthritis Index (WOMAC) score improved after injection in the high-dose group over 6 months. Moreover, arthroscopy and MRI confirmed that the cartilage defect size decreased while the cartilage volume increased in the high-dose group, additionally, histology confirmed thick, hyaline-like cartilage regeneration. Their findings demonstrated that intra-articular injection of high dose (1.0 × 10^8^ ) AD-MSCs into the OA knee improved function and relieved pain of the knee joint without causing adverse events, and retarded cartilage degeneration by regeneration of hyaline-like articular cartilage [66]. Evidence from Jaskarndip Chahal et al.’s [80] study showed that autologous bone marrow mesenchymal stromal cells (BM-MSCs) were safe and resulted in significant improvements in patient-reported outcome measures (PROMs). In their study, advanced knee OA patients received a single intra-articular injection of 1, 10, or 50 million BM-MSCs for 12 months. The 50 million doses achieved clinically relevant improvements across most PROMs, there were significant overall improvements in Osteoarthritis Outcome Score pain, symptoms, quality of life, and WOMAC stiffness relative to baseline. Moreover, pro-inflammatory monocytes/macrophages and interleukin 12 levels decreased in the synovial fluid after MSC injection at the 50 and 10 million doses. Most importantly, although there was no direct protective effect of MSCs on cartilage regeneration by MRI testing, they had shown that BM-MSCs, accompanied by the elevated levels of anti-inflammatory and antifibrotic gene and protein markers, which were likely to have improved clinical efficacy in terms of PROMs over 12 months. They concluded that BM-MSCs reduced synovial inflammation in OA [80]. The study by Mohsen Emadedin et al. [81] investigated the safety of treatment with BM-MSCs transplanted in patients with OA of the knee, ankle, or hip. In their study, they showed that all subjects had no serious adverse events, such as pulmonary embolism, death, or systemic complications. There were very minor localized adverse effects such as rash and erythema in a limited number of patients. The results showed that the injection of MSCs in different OA-affected joints was safe and therapeutically beneficial [81]. These findings provided robust evidences that clinical outcomes such as pain and function were improved after the application of intra-articular MSCs. However, in randomized controlled trials, there were controversial results in clinical outcomes [82, 83], One study reported that there was no significant change from baseline to final follow-up in the MSC group and that there was no difference between groups in terms of the WORMS score [82]. In Wakitani et al.’s [83] study, they showed that the clinical improvement was not significantly different among groups, however, the cartilage repair was better in the MSC group than in the cell-free control group. Clinical trials with MSCs for the treatment of joint diseases are summarized in Table 1 and the tissue repair mechanism was shown in Fig. 1. Additional researches need to be done for evaluating the impact of MSCs in the knee OA.

**Table 1:** Details of the papers describing clinical trials with MSCs for the treatment of joint diseases

References	Cell types/source	Applications	Delivery intervention	Study type	Patient No.	Results	
						Adverse	Beneficial
[84]	hBM-MSC	kOA	Injection of 20–24×10^6^ cells	Original article	6	One year, no local or systemic adverse events	Increase: functional, thickness, repair tissue over the subchondral bone Decrease: edematous and pain
[70]	hADSCs	CMP	The ADSCs along with PRP, hyaluronic acid, and CaCl_2_	Case report	3	18 months, all three patients did not report any serious side effects	Increase: the damaged tissues (softened cartilages) Decrease: pain
[85]	hUCB-MSCs	Osteochondral defect	Injection of MSC (5 × 10^6^/ml) in HA (4%)	Case report	1	5.5 years, no specific adverse reactions	Increase: IKDC, WOMAC, cartilage-like aspect, GAGs, Collagen type II Decrease: VAS and collagen type I, no bone formation
[86]	hUCB-MSCs	OA	Injection of MSC (1.15/1.25 × 10^7^ or 1.65/2 × 10^7^) in HA	Open-label, single-arm, phase I/II	7	7 years, no cases of osteogenesis or tumorigenesis; mild to moderate treatment-emergent adverse events	Increase: IKDC and aspect of hyaline-like cartilage Decrease: VAS
[87]	hUC-MSCs	KOA	Injection of UC MSC (20×10^6^) once or twice vs HA injection	Randomized double-blind, controlled phase I/II	29	No serious AEs, deaths, permanent disability, neoplasia, or septic arthritis cases; acute synovitis and mild to moderate symptomatic effusion	Increase: WOMAC, decrease: VAS, pain, and disability
[80]	hBM-MSCs	KOA	Injection of BM MSCs (1 × 10^6^/10 × 10^6^/50 × 10^6^)	Nonrandomized, open-label, dose-escalation phase I/II clinical trial conducted	12	12 months, no serious adverse events; minor, transient adverse events	Improvement in KOOS pain, symptoms, quality of life, and WOMAC stiffness
[88]	hUCB-MSCs	OA	Injection of hUCB-MSC (7.5 × 10^6^) in HA(4%)-Commercial	Retrospective case series	128	2 years, no adverse reactions or postoperative complications were noted	Increase: IKDC, ,WOMAC, and MOCART Decrease: VAS
[89]	hUC-MSCs	KOA	Injection of wjMSC (10 × 10^6^) in 2 ml secretome + 2 ml HA	Open-label, single-arm, phase I/II	29	3.5 years, no adverse reactions are reported	Increase: IKDC and WOMAC Decrease: VAS

## Conclusion

CMP is a common disease of the PFJ. As a non-invasive diagnosis method, MRI plays an indispensable role in evaluating the severity of CMP. For patients, conservative intervention is definitely a better choice than surgery, but whether it can promote the self-repair of patellar cartilage is questionable, making the long-term efficacy difficult to guarantee. Nevertheless, it was difficult to accurately conclude as to the effectiveness of MSCs on clinical outcomes and cartilage repair in cartilage lesions. Fortunately, large quantities of reports have shown that the beneficial effect of MSCs on cartilage regeneration and with better clinical outcomes. The direct intra-articular injection of MSCs could offer great advantages if it could be translated into clinical practice as it could avoid surgeries and associated side effects thus would be a better modality for the treatment of CMP.

## Data Availability

Not applicable.

## Abbreviations

CMP: Chondromalacia patellae
AKP: Anterior knee pain
MRI: Magnetic resonance imaging
MSCs: Mesenchymal stem cells
PF: Patellofemoral
TF: Tibiofemoral
LPCS: Lateral patellar compression syndrome
OA/KOA: Osteoarthritis/Knee osteoarthritis
ACI: Autologous chondrocyte implantation
MACI: Matrix-assisted chondrocyte implantation
ESC-MSCs: Embryonic stem cell-induced mesenchymal stem cells
haMSCs: Human adipose-derived mesenchymal stem cells
AD-MSCs: Adipose tissue-derived MSCs
BM-MSCs: Bone marrow mesenchymal stromal cells
UCB-MSCs: Umbilical cord blood-derived mesenchymal stem cells
PROMs: Patient-reported outcome measures
WOMAC: Western Ontario and McMaster Universities Osteoarthritis Index
HA: Hyaluronic acid
IKDC: International Knee Documentation and Committee score
GAGs: Glycosaminoglycans
VAS: Visual analog scale
MOCART: Magnetic Resonance Observation of Cartilage Repair Tissue
